# Dynamics of a Pregnancy When Two Become Four: A Case Report and Literature Review

**DOI:** 10.7759/cureus.873

**Published:** 2016-11-11

**Authors:** Murid Javed, Nareman Altorairi, Hamad Alsufyan

**Affiliations:** 1 Embryology and Andrology Laboratories, Thuriah Medical Center; 2 Reproductive Endocrinology and Infertility, Thuriah Medical Center

**Keywords:** monozygotic twins, pregnancy complications, multiple gestation, fetal reduction

## Abstract

The incidence of pregnancies with multiple gestational sacs has increased with the use of assisted reproductive technology because more than one embryo is frequently transferred. The splitting of one or more embryos further complicates the pregnancy. Some of these complications include intrauterine fetal death, growth restriction, discordant birth weight, and preterm delivery. Monozygotic twins suffer from a few unique complications including twin-twin transfusion syndrome, twin reversed arterial perfusion sequence, and twin anemia-polycythemia sequence. Therefore, patients should be informed about the possible obstetric complications regarding monozygotic twinning after embryo transfer as poor obstetric/perinatal outcome is significantly impacted by the presence of an "extra" fetus. The etiology of monozygotic twinning is not fully understood although a few risk factors have been identified. The objective of this communication is to report successful management of a pregnancy resulting in four gestational sacs after transfer of two embryos on day-three post retrieval.

## Introduction

The widespread use of assisted reproductive technologies has led to an increase in the prevalence of monozygotic twins [1]. Monozygotic twins, triplets or quadruplets result from the splitting of a single fertilized embryo at the early stage of embryo development to form two or more separate genetically identical embryos. In assisted reproduction, usually more than one embryo is frequently transferred; however, the subsequent splitting of one or more embryos results in high-order multiple gestation of three or more fetuses. The incidence of monozygotic twinning after single embryo transfer has been reported to be lower for day two-three transfers than for day five-six transfers: 1.71% vs 2.50%, respectively [2]. The risk factors for increase in monozygotic twinning include assisted hatching for day two-three transfers and having one or more prior pregnancies for day five-six transfers [2]; < 36 yrs age, extended embryo culture [3]; zona pellucida structure and zona/embryo manipulation [4].

Monozygotic twinning is universally accepted as a postfertilization event resulting from the splitting of the embryo during its first two weeks of development. The stage at which splitting occurs determines chorionicity and amnionicity [5]. When two fetuses share the same placenta a monochorionic pair results. Twinning after blastocyst transfer results in monochorionic placentation [1]. 

Triplets complicated by a monochorionic pair are at further increased risk compared with trichorionic/triamniotic triplets, including a higher likelihood of intrauterine fetal death, growth restriction, discordant birth weight, and preterm delivery. The selective reduction of a monochorionic pair is frequently offered in the obstetric management of triplets and higher-order multiples because gestational length is prolonged, particularly with reduction of the monochorionic pair [6]. The objective of this report is to describe dynamics of a pregnancy that resulted in four gestation sacs after transfer of two embryos on day-three post retrieval. Informed consent was obtained from the patient for this study.

## Case presentation

A 32-year-old female with a history of polycystic ovary (PCO) underwent a third intracytoplasmic sperm injection (ICSI) attempt. On the first ICSI cycle, she achieved biochemical pregnancy. Her second ICSI attempt resulted in first trimester miscarriage. Before starting the third ICSI cycle, her elevated prolactin was corrected by one weekly 0.5 mg tablet of Dostinex (cabergoline, Pfizer Inc). She received 50 microgram thyroxine daily. Her thyroid-stimulating hormone (TSH), physical examination, and hysterosalpingogram (hSG) were normal. The partner had normal semen parameters. The ovarian stimulation was initiated on day-three of the cycle with 150 IU Gonal–F (recombinant follitropin *alfa, *Merck Biopharma, MA, USA) for six days with a dose increase to 225 IU for another two days. From day eight to 11 of the cycle, 0.25 mg daily injection of gonadotrophin-releasing hormone (GnRH) antagonist, Cetrotide (cetrorelix acetate, Merck Biopharma) was administered. Final oocyte maturation was achieved by a single injection of 10,000 IU of human chorionic gonadotropin (hCG). The oocytes were retrieved 36 hours post hCG injection.

At retrieval, 13 oocytes were collected: three metaphase II (MII), seven germinal vesicle (GV) and three empty zona. Oocytes denudation, semen processing, ICSI and culture were performed using standard protocols. Only three MII oocytes were injected. A fertilization check 18 hours post ICSI revealed two normally fertilized oocytes and the third had only one pronucleus. On day-three post retrieval, two embryos—six cells grade two and seven cells grade two were transferred under transabdominal ultrasound guidance. Assisted hatching was performed on both transferred embryos. Luteal support was provided by Cyclogest pessaries (L.D.Collins and CO, UK).

**Figure 1:**
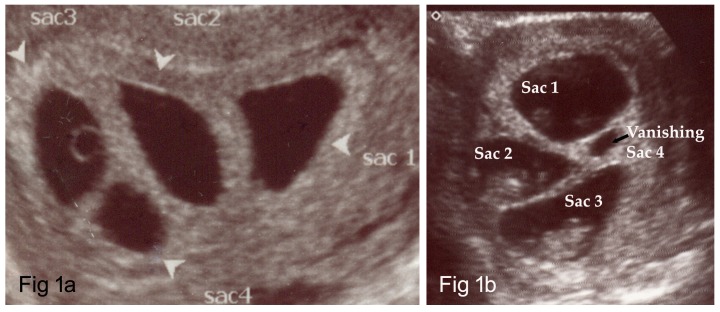
An ultrasound image showing four gestational sacs (Fig 1a) and a vanishing sac 4 (Fig 1b)

The first pregnancy test two weeks post embryo transfer had an hCG level of 2714 mIU/mL which increased to 26862 mIU/mL six days later. An ultrasound scan at five weeks revealed four intrauterine gestational sacs (Figure 1). Later, a monochorionic pregnancy with four distinct gestational sacs and three embryos with cardiac activity was confirmed. The measurements for sacs one–four were 1.96 x 0.676, 1.70 x 0.83, 1.33 x 0.794, and 0.541 x 0.451 cm, respectively. A minor bleed 9 x 7 x 7 mm was noted on the anterio-lateral of sac one. The heart activity was noted in sacs one, two, and three but not in sac four.

Another ultrasound scan at seven weeks and two days confirmed triplet pregnancy with heart rates 145, 154, and 150 BPM for sacs one, two, and three, respectively. No fetal pole or yolk sac was noted in sac four. The crown-rump lengths (CRL) at eight weeks, six days were 2.35, 2.35, and 2.06 cm and heart rates were 173, 178, and 183 BPM for sacs one, two, and three, respectively. The risks of monozygotic pregnancies, medical abortion, and selective embryo reduction were extensively discussed with the couple. This patient had experienced spontaneous abortion at 16 weeks in a previous pregnancy, therefore cervical cerclage was offered.

The couple accepted fetal reduction and cervical cerclage. At 10 weeks and two days, twin viable pregnancy was confirmed with CRL 3.45 and 3.28 cm and heart activity 164 and 160 BPM, respectively for sac one and two.

**Figure 2:**
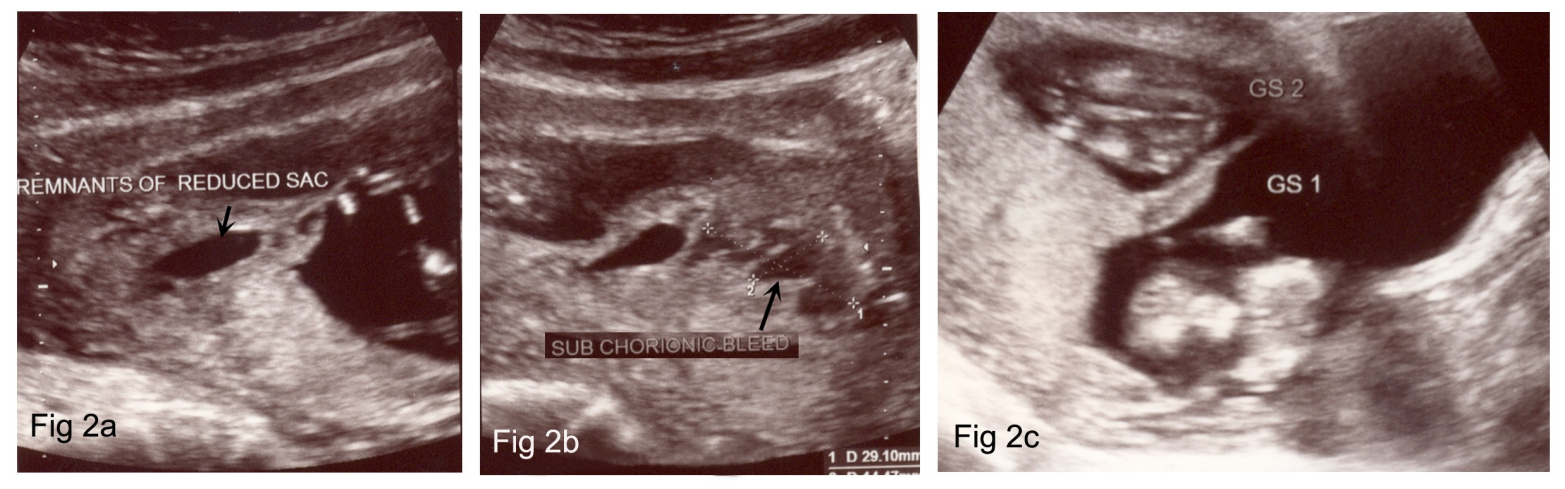
An ultrasound image showing remnants of reduced fetal sac (Fig 2a), sub-chorionic bleed (Fig 2b) and two fetal sacs resulting in twin delivery (Fig 2c)

A 23 x 5 mm subchorionic bleed was noted posterio-lateral of sac two. The remnants of the reduced fetal sac were still noted measuring 28 x 15 mm. Estimated fetal weights for sac one and two at 12 weeks and six days were 57 and 70 g, respectively. Amniotic fluid volumes were adequate. The healthy twin boys were vaginally delivered after 36 weeks gestation.

## Discussion

In assisted reproduction, usually more than one embryo is frequently transferred, subsequently a single embryo dividing into two can be a high-order multiple gestation of three or more fetuses. The incidence of monozygotic twinning after elective single embryo transfer among women undergoing assisted reproductive technology (ART) was 1.71 to 2.50% in USA depending on the day of embryo transfer; however, the overall incidence after a single embryo transfer was 2.24% during the years 2003–2012 [2]. 

The etiology of monozygotic twinning is uncertain. The risk factors include blastocyst transfer [2], younger maternal age [3], higher frequency of ovulatory disorders, lower frequencies of diminished ovarian reserve, prior pregnancies, and assisted hatching [2]. The association of assisting hatching with the monozygotic twinning is contradictory. A report [7] indicated that assisted hatching increased risk of twinning; however, another report [8] did not find any difference between zona manipulated and zona intact groups. A case of monochorionic quadamniotic pregnancy after day four transfer of a single embryo and another case of monochorionic triamniotic pregnancy after transfer of a single blastocyst resulting from conventional in vitro fertilization (IVF) have been reported [9]. In the former case, the embryo underwent single cell biopsy at 10 cell stage indicating that zona or embryo manipulation may be a cause. Pregnancies with ≥3 fetuses resulting from a single embryo are exceedingly rare. In this report two embryos were transferred and subsequently each embryo divided into two resulting in four gestational sacs.

Unique complications such as twin-twin transfusion syndrome, twin reversed arterial perfusion sequence, twin anemia-polycythemia sequence, selective intrauterine growth restriction and in utero death of one twin pose significant risks for death or severe neurologic morbidity in the co-twin [10]. This can be easily and accurately accessed with first trimester ultrasound by evaluating the interface of the intertwin membrane with the placenta. This should now be the standard of care for all multiple gestations. Also, patients should be informed about the possible obstetric complications regarding monozygotic twinning after embryo transfer as poor obstetric/perinatal outcome is significantly impacted by the presence of an "extra" fetus. In the present case report, the fourth gestational sac vanished naturally and to avoid triplets' complication, fetal reduction was performed. Two healthy monozygotic twin males were delivered after 36 weeks gestation. For women aged < 36 years, elective single blastocyst transfer is recommended to reduce multiple pregnancy gestation [3].

## Conclusions

A pregnancy with four gestational sacs can be managed successfully by regular scanning, monitoring the patient’s medical condition, and fetal reduction. As the factors causing splitting are not fully understood, patients should be informed of the risks of monozygotic pregnancies after embryo transfer.
